# The Hypothiocyanite and Amantadine Combination Treatment Prevents Lethal Influenza A Virus Infection in Mice

**DOI:** 10.3389/fimmu.2022.859033

**Published:** 2022-05-18

**Authors:** Nuha Milad Ashtiwi, Demba Sarr, Tamás Nagy, Z. Beau Reneer, Ralph A. Tripp, Balázs Rada

**Affiliations:** ^1^ Department of Infectious Diseases, College of Veterinary Medicine, University of Georgia, Athens, GA, United States; ^2^ Department of Pathology, College of Veterinary Medicine, University of Georgia, Athens, GA, United States

**Keywords:** hypothiocyanite, amantadine, treatment, lactoperoxidase, lethal, influenza

## Abstract

The influenza virus has a large clinical burden and is associated with significant mortality and morbidity. The development of effective drugs for the treatment or prevention of influenza is important in order to reduce its impact. Adamantanes and neuraminidase inhibitors are two classes of anti-influenza drugs in which resistance has developed; thus, there is an urgent need to explore new therapeutic options. Boosting antiviral innate immune mechanisms in the airways represents an attractive approach. Hypothiocyanite (OSCN^−^) is produced by the airway epithelium and is effective in reducing the replication of several influenza A virus strains *in vitro*. It remains, however, largely unexplored whether OSCN^−^ has such an antiviral effect *in vivo*. Here we determined the therapeutic potential of OSCN^−^, alone or in combination with amantadine (AMT), in preventing lethal influenza A virus replication in mice and *in vitro*. Mice intranasally infected with a lethal dose of A/Puerto Rico/8/1934 (H1N1) or A/Hong Kong/8/1968 (H3N2) were cured by the combination treatment of OSCN^−^ and AMT. Monotherapy with OSCN^−^ or AMT alone did not substantially improve survival outcomes. However, AMT+OSCN^−^ treatment significantly inhibited viral replication, and *in vitro* treatment inhibited viral entry and nuclear transport of different influenza A virus strains (H1N1 and H3N2) including the AMT-resistant strain A/WSN/33 (H1N1). A triple combination treatment consisting of AMT, oseltamivir, and OSCN^−^ was also tested and further inhibited *in vitro* viral replication of the AMT-resistant A/WSN/33 strain. These results suggest that OSCN^−^ is a promising anti-influenza treatment option when combined with other antiviral drugs.

## Introduction

The influenza virus can cause some mortality and high morbidity rates, especially in immunocompromised patients and those with comorbidities such as diabetes, chronic renal failure, malignancies, or immune system disorders (1–4). Four types of anti-influenza drugs having different mechanisms of action are available. Amantadine (AMT) and rimantadine are viral M2 channel inhibitors, and oseltamivir (OSL) and zanamivir are neuraminidase inhibitors (NAIs). The NAIs are only effective if the medication is administered within 48 h of symptom onset, and delayed treatment can result in high morbidity and mortality rates and a reduction in therapeutic efficacy (5, 6). Insufficient inhibition of the viral replication leads to the emergence of drug resistance, which is associated with prolonged disease duration (7, 8). Early published results showed that some 2009 H1N1 virus strains are AMT-resistant, which is associated with an S31N mutation in the M2 proton channel, but they remain susceptible to NAIs, i.e., OSL and zanamivir (9). It has been reported that many H1N1 isolates tested in the United States are resistant to OSL (9, 10). As almost all seasonal H3N2 and H1N1 viruses are resistant to AMT, and currently the majority of H1N1 strains from clinical isolates are also resistant to OSL, there is a strong rationale for developing new, combination therapies.

A previous study showed that therapeutic treatment with AMT, ribavirin, and/or OSL against a panel of influenza A viruses (IAVs) in triple combination had greater effects than any of the double combinations (11). A triple combination of anti-influenza drugs (TCAD) was shown to be effective against several AMT-resistant and OSL-resistant influenza viruses (9). AMT and OSL were combined at doses that were clinically approved, and these concentrations had less therapeutic efficacy when administered as a monotherapy (9). In addition, other studies also showed clinical efficacy of TCAD therapy in mice and that TCAD therapy increased the survival rate and lowered the body weight loss of mice infected with susceptible and AMT-resistant A/H1N1 viruses (9). These results suggested that the combined treatment is an effective, anti-influenza approach to overcome drug resistance issues.

Recently, we have reported that Dual oxidase 1 (Duox1), an NADPH oxidase family member highly expressed in the respiratory epithelium, promotes antiviral innate immunity in IAV-infected mice (12). *Duox1^−/−^* mice infected with IAV had enhanced mortality and morbidity and impaired lung viral clearance (12). These data support a previously proposed model (13) where Duox1 generates intraluminal H_2_O_2_ that is used by lactoperoxidase (LPO) to convert its main substrate thiocyanate (SCN^−^) to antimicrobial hypothiocyanite (OSCN^−^) (12). We have shown that several influenza A or B strains are inactivated *in vitro* by OSCN^−^ generated by LPO, SCN^−^, and H_2_O_2_ (12, 14, 15). H_2_O_2_ is derived from an enzymatic reaction in a cell-free system or by Duox1 present in normal human bronchial epithelial (NHBE) cells (12, 14, 15). We also showed that OSCN^−^ diminished influenza replication and viral RNA synthesis in host cells that were inhibited by the H_2_O_2_ scavenger, catalase (12). The binding of influenza viruses to host cells and viral entry were both significantly reduced by OSCN^−^ in an H_2_O_2_-dependent manner *in vitro* (12). Overall, this novel *in vivo* antiviral function of OSCN^−^ and Duox1 proposes that enhancing this mechanism could have therapeutic potential in treating influenza infections.

In this study, we evaluated the therapeutic potential of OSCN^−^ single treatment and the OSCN^−^+AMT combination treatment on lethal influenza infection in a mouse model. We also studied the effects of these treatments on viral uptake and replication in host cells. Our data show that combination therapy of OSCN^−^+AMT prevents mortality in lethal IAV infection *in vivo* by significantly reducing viral replication in host cells. Our data present an innovative approach to treating severe influenza infection that combines a traditional antiviral drug with a natural product of the innate immune system and achieves synergistic effects in viral clearance and prevention of influenza-related morbidity and mortality.

## Materials and Methods

### Influenza Virus Propagation

IAVs were purchased from BEI Resources (managed by the American Type Culture Collection [ATCC]), NIH. The following IAVs were used in this study: A/California/04/2009 (H1N1; NR-13663), A/Puerto Rico/8/1934 (H1N1; NR-3169), A/turkey/Kansas/4880/1980 (H1N1; NR-3473), A/WSN/1933 (H1N1;(NR-3688), A/Aichi/2/1968 (H3H2; NR-3177), A/Wisconsin/67/2005 (H3N2; NR-41800), and A/Hong Kong/8/1968 (H3N2; NR-346). A/Texas/50/2012 (H3N2) was received through the Center for Research on Influenza Pathogenesis (CRIP). IAVs were propagated in Madin–Darby canine kidney (MDCK) cells as described (12). Briefly, viruses were cultured in MDCK cells (ATCC, CCL034) using an infection medium (DMEM/F-12 supplemented with 1 mM of l-glutamine with 1 μg/ml of tosylsulfonyl phenylalanyl chloromethyl ketone-treated trypsin). Viruses were harvested 48 h post-infection (hpi). A/Puerto Rico/8/1934 (H1N1) was grown in the allantoic cavity of 9-10-day-old specific pathogen-free embryonated chicken eggs at 37°C. Viral titers were determined by plaque-forming unit (PFU) assay and hemagglutination (HA) assay (12). The AMT-resistant influenza A/WSN/1933 virus was first passaged in MDCK cells, then grown in 10-day-old embryonated chicken eggs for 2 days, and titrated by plaque assay. The mouse-adapted A/Hong Kong/8/1968 H3N2 virus was passaged in MDCK cells. A/California/04/2009 (H1N1), A/Aichi/02/1968 (H3N2), A/Texas/50/2012 H3N2, and A/Wisconsin/67/2005 H3N2 were passaged in MDCK cells once before *in vitro* assays.

### Animal Experiments

All mouse procedures were approved by the Animal Care and Use Committee at the University of Georgia (UGA; protocol ID: A2017 07-010-Y2-A1). Six-week-old C57BL/6 mice from Jackson laboratories were anesthetized by intraperitoneal injection of 2,2-tribromoethanol (Avertin) at the dose of 200 mg/kg. Mice were infected intranasally with a 50-µl suspension of 150 PFU of A/Puerto Rico/8/1934 influenza virus and 3,000 PFU of A/Hong Kong/8/1968 influenza virus. Each mouse received an infectious dose of 2 × LD_50_ to achieve 100% lethality. Influenza virus lethal dose 50 (LD_50_) lung titers were determined using the method of Reed and Muench (16): log_10_ 50% endpoint dilution = log_10_ of dilution showing mortality next above 50% − (difference of logarithms × logarithm of the dilution factor). For all experiments, treatments were begun 48 h after infection and administered twice a day by oral gavage without anesthesia. A 5-day treatment of either monotherapy or a combined drug regimen was administered to mice given a lethal A/PR/8/1934 or A/Hong Kong/8/1968 infection in two independent experiments. Mice receiving a lethal infection of A/Hong Kong/8/1968 were also treated for 10 days. For all studies, AMT 46 mg/kg/day and OSL 25 mg/kg/day were administered alone or in combination. The projected pharmacokinetic parameters were expected to yield plasma levels in mice similar to those in humans (17). Following infection and during the treatment period, mice were monitored daily for 21 days for survival and weight loss. Mice with severe symptoms having a body weight loss of ≥30% were euthanized. For endpoint studies, mice were euthanized, and their lungs were removed at indicated times for lung histology and bronchoalveolar lavage fluid (BALF) studies. BAL samples were collected by injecting 1 ml of sterile PBS into the lung as previously described (12). Collected BALF was separated by centrifugation (400*g*, 10 min) into BAL cells (pellet) and cell-free BAL supernatants. BAL cells were used to measure influenza mRNA levels by qPCR, while BAL supernatants were used to determine viral titers by plaque assays.

### Antiviral Compounds

AMT and OSL phosphate were obtained from Sigma-Aldrich (St. Louis, MO, USA). All drugs were dissolved in sterile water as previously reported (18, 19). Drugs were administered in sterile water *via* oral gavage. Double and triple combinations were co-formulated and administered as a single solution. The placebo (sterile water) was administered in parallel with antiviral treatments in the same volume (100 µl).

### OSCN^−^ Production and Administration

To generate OSCN^−^ *in vitro*, components of the LPO-based antiviral system were used as previously reported (12, 14). Physiologically relevant concentrations were used, i.e., 6.5 μg/ml of LPO, 400 μM of SCN^−^, 0.005 M of glucose, and 0.01 U/ml of glucose oxidase (12, 15). The entire solution containing freshly produced OSCN^−^ was administered alone or in combination with AMT or OSL twice daily by oral gavage (100 µl) according to the experimental protocols.

### Histopathology

Murine lungs and tracheas after 5 days post-infection (dpi) (3 days after treatment) with A/PR/8/34 or A/HK/8/68 were inflated with 1 ml of 10% neutral buffered formalin and harvested as described (12). All collected tissues were consistently fixed overnight in neutral formalin. Tissue samples were processed, and sections were prepared by the Histology Laboratory at the College of Veterinary Medicine at UGA. Sections were either left unstained for subsequent experiments (immunofluorescence) or stained with H&E as described (12).

### Cytotoxicity Assays

The potential cytotoxicity of the tested antiviral compounds was evaluated using the MTT Cell Proliferation Kit (OZ Biosciences, Catalog # MT01000). Briefly, 100,000 MDCK cells in DMEM media were seeded per well of 96-well cell culture plates using eight wells for each compound. Twenty-four hours later, the growth medium was removed, washed at 37°C, and preheated, with sterile phosphate-buffered saline (PBS) and then 200 µl of fresh DMEM [no fetal bovine serum (FBS)] medium containing the compounds. The compounds were tested at concentrations used throughout the manuscript of viral infection studies, i.e., OSL phosphate at 5 μg/ml and AMT at 15 μg/ml. Untreated, influenza virus-infected cells were used as a positive control for cell damage, and uninfected, untreated cells were used as a negative control. The virus-infected, untreated cells exhibited 100% cytopathology at 3 dpi. For single and double combination treatments, each drug was tested at the same concentrations in eight replicates per experiment. The cytotoxicity of single and double drug combinations was determined using the same experimental protocol in three separate experiments and using the same concentration ranges 3 days after drug addition. The MTT Cell Proliferation kit is a colorimetric assay measuring the mitochondrial reductase activity in living cells that reduces MTT to formazan dyes, giving a blue/purple color. It is based on the cleavage of the membrane-permeable yellow tetrazolium salt MTT to formazan crystals by metabolically active cells. A solubilization solution is then added to dissolve formazan into a colored solution. Quantification of cell viability was determined by absorbance at 570 nm using spectrophotometry and expressed as a percentage of 100% cytotoxicity.

### Cell Cultures

MDCK (CCL34™) cells were maintained under humidity at 37°C with 5% CO_2_ in Dulbecco’s modified Eagle’s medium/Nutrient mixture F-12 Ham (DMEM/F-12) (MilliporeSigma, St. Louis, MO, USA) supplemented with 10% heat-inactivated FBS (Gemini Bio-Products, West Sacramento, CA, USA; Catalog # 100-106). Primary NHBE cells were purchased from Lonza (Walkersville, MD, USA). To seed NHBE cells, 24-well polyester (0.4-μm pore) membrane transwells (Costar) were used at a density of 5 × 10^4^ cells/well. When the NHBE cells reached confluency, the B-ALI™ growth medium in the upper chamber was removed, and the B-ALI™ growth medium in the lower chamber was replaced with the B-ALI™ differentiation medium (Lonza, Walkersville, MD, USA). Cells were kept on the air–liquid interface (ALI) for 4 weeks by feeding every other day with an ALI differentiation medium (the surface of ALI cultures was washed with sterile Hanks’ Balanced Salt Solution (HBSS) every other day). Antibiotics (penicillin and streptomycin; Life Technologies, Grand Island, NY, USA) were added to the media up to 4 days before experiments, as previously described (14, 20). NHBE cells were washed three times with PBS to remove excess mucus on the apical surface prior to infection. Viruses added to the apical side of NHBE cells were allowed to adsorb for 1 h at 37°C. Unbound viruses were removed by aspiration, and NHBE cells were washed again with PBS 3 sequential times. NHBE cells were incubated for the indicated times post-infection at 37°C. Viruses released apically were harvested by the apical addition, and a collection of 300 µl of 0.05% BSA-BEBM that was allowed to equilibrate at 37°C for 30 min. Samples were stored at −80°C until assayed.

### Plaque Assays

Plaque assays were performed as described (12, 14, 15). Briefly, A/Puerto Rico/8/1934, A/California/04/2009, A/WSN/1933, A/Aichi/02/1968, A/Texas/50/2012, A/Hong Kong/8/1968, or A/Wisconsin/67/2005 were used to infect MDCK cells or NHBE cells in the presence or absence of treatment compounds to evaluate their antiviral activity. A confluent monolayer of MDCK or NHBE cells was inoculated with 100 multiplicity of infection (MOI) viruses in DMEM with 10% bovine serum albumin for 1 h at 4°C and then 37°C for 1 h. The cells were washed with PBS after inoculum removal. The cells were then overlaid with DMEM containing 1.2% Avicel and *N*-acetyl trypsin (2.0 μg/ml). To observe the effect of the compounds on plaque formation, the overlay media were supplemented with compounds at testing concentrations. The monolayers were fixed and stained with crystal violet dye solution (0.2% crystal violet and 20% methanol) to count plaques. Viral titers were calculated using 10-fold serial dilutions as before (12, 14, 15). The sizes of representative plaques were determined by measuring the average diameter of the plaques in two directions that are perpendicular to each other. Plaque size is expressed in mm.

### Influenza Virus Entry Assays

Indirect immunofluorescence techniques were performed to detect the effect of different treatments at different steps of the influenza virus replication cycle using a Zeiss confocal microscope. MDCK cells were propagated as described (12) at 37°C for 24 h and infected at MOI = 100 at 4°C for 1 h for evaluating nucleoprotein (NP) expression as described (12). Unbound virus was removed by washing three times with cold Dulbecco’s PBS (DPBS). Cells were re-fed with OPTI-PRO serum-free medium, then treated with treatments or 0.5% dimethyl sulfoxide (DMSO), and incubated at 37°C. To monitor NP expression, infection was stopped with 4% paraformaldehyde (PFA) at different time points. At 6 hpi, cells were fixed, and NP expression was determined at 24 hpi.

### Immunofluorescence Assays

Detection of influenza viral NP, M1, and M2 proteins in MDCK cells under different treatment conditions was performed as described (12). Viral entry was determined in MDCK cells at 24 hpi for cells infected with the following H1N1 and H3N2 influenza strains (MOI of 100) at 4°C for 1 h: A/California/04/2009, A/Puerto Rico/8/1934, A/Aichi/2/1968, A/Texas/50/2012, A/Wisconsin/67/2005, and A/Hong Kong/8/1968. Unbound viruses were removed by washing host cells three times with cold PBS. Cells were refed with MDCK media and incubated at 37°C. Cells were stained for the viral M1, M2, and NP proteins as described (12). Cells were treated with AMT and/or OSL at 15 and/or 5 μg/ml, respectively. To monitor the uncoating and nuclear import processes, infection was stopped with 4% PFA at 3 and 6 hpi, respectively. Immunofluorescence assays (IFAs) were conducted using influenza M1- and M2-specific monoclonal antibodies as described (12). Briefly, cells were incubated with anti-IAV NP antibody (AA5H, Abcam, Waltham, MA, USA), influenza A M1 monoclonal antibody (GA2B) (MA1-80736), and influenza A M2 monoclonal antibody (14C2) (MA1-082) for 12 h at 4°. Then cells were incubated for 1 h with an Alexa 488-conjugated goat anti-mouse IgG (H + L) secondary antibody (A32723) and counterstained with 4′,6-diamidino-2-phenylindole nuclear stain (DAPI). The images were acquired at ×60 magnification using a confocal microscope. The immunofluorescence was quantified using Fiji software (NIH, Bethesda, MA, USA) for each fluorescent channel. The fluorescent intensities of studied viral markers (M1, M2, and NP) were normalized on the DNA of MDCK cells and are expressed as M1/DNA, M2/DNA, or NP/DNA ratios.

### Quantitative Real-Time PCR

RNA extraction from treated MDCK, mouse BAL cells, or NHBE cells was performed using TRIzol (Catalog # 15596018; Invitrogen/Thermo Fisher Scientific, Carlsbad, CA, USA). cDNA was synthesized from 2 μg of total RNA using the High-capacity Reverse Transcriptase cDNA kit (Catalog # 4368814; Applied Biosystems/Thermo Fisher Scientific, Carlsbad, CA, USA). Each cDNA sample was diluted 10-fold, and real-time PCR was performed using SYBR Green PCR Master Mix (Catalog # 4309155; Applied Biosystems/Thermo Fisher Scientific, Carlsbad, CA, USA). The primer sequences used for gene amplification are listed as follows: β-actin forward: 5′‐CCAACCGCGAGAAGATGA‐3′, β‐actin reverse: 5′‐ CCAGAGGCGTACAGGGATAG‐3′. The forward NR-15594 and reverse NR-15595 16 primers used to amplify H1N1 and H3N2 transcripts were from the NR-15592 kit (Influenza Virus Real-Time RT-PCR Assay, BEI Resources, ATCC, NIH).

### Statistics

The data are presented as mean ± SEM or mean ± SD, as indicated in the figure legends. Statistical significance between experimental groups was determined using chi-Square, two-tailed Student’s t-test, or Mann–Whitney U-test. Significance between multiple groups was assessed using either one-way or two-way ANOVA with Kruskal–Wallis followed by Dunn’s multiple comparisons. For each test, p < 0.05 was considered significant.

## Results

### The OSCN^−^/AMT Combination Treatment Reduces Influenza A Virus Replication *In Vitro*

**Figure 1:**
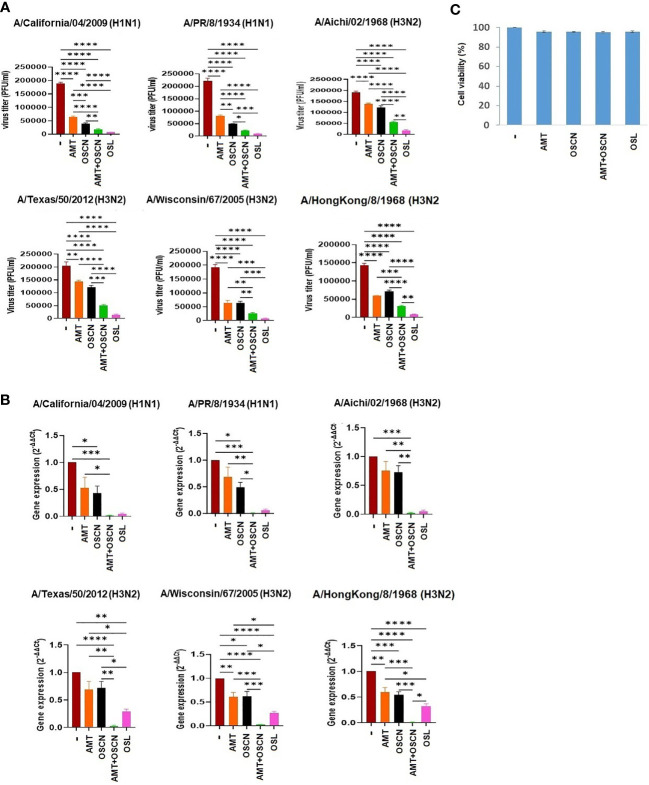
The AMT+OSCN^−^ combination treatment significantly reduces the replication of several influenza A virus strains *in vitro*. **(A)** Confluent monolayers of MDCK cells were inoculated with 100 MOI of the indicated six H1N1 and H3N2 IAV strains individually. The overlay media were supplemented with the indicated antiviral compounds. Viral proliferation was measured by PFU assay. OSL phosphate was used as a control. Data are mean ± SEM of n = 6 (A/California/04/2009, A/PR/8/34, and A/Aichi/2/1968), n = 4 (A/Texas/50/12 and A/Hong Kong/8/68), and n = 5 (A/Wisconsin/67/2005) independent experiments. One-way ANOVA and Tukey’s multiple comparisons tests. **(B)** Viral mRNA levels were measured under the exact same conditions and at the same time as in panel **(A)** by qRT-PCR for the indicated viral strains. All shown results were normalized and compared to housekeeping genes as control. OSL was used as positive control. Mean ± SEM, n = 3. One-way ANOVA and Tukey’s multiple comparisons test. **(C)** MDCK cells were exposed to the indicated treatment options without viral infection, and cell toxicity was measured by the MTT Cell Proliferation Kit (colorimetric assay). At 3 days after drug addition, the treatment was added at maximum dose: OSL at 5 μg/ml and AMT at 15 μg/ml. Untreated but infected cells were used as positive control for cell toxicity, while uninfected, untreated cells were used as negative control (n = 3). AMT, amantadine; MOI, multiplicity of infection; OSCN^−^, hypothiocyanite; OSL, oseltamivir; PFU, plaque-forming unit; MDCK, Madin–Darby canine kidney; IAV, influenza A virus.*, p < 0.05; **, p < 0.01; ***, p < 0.001; ****, p < 0.0001.

We have previously shown that OSCN^−^ inhibits influenza virus replication *in vitro* (12, 14, 15). We sought to determine whether combining OSCN^−^ with AMT drug treatment would further increase antiviral efficacy. Six influenza A virus strains were tested *in vitro* on MDCK cells at a high MOI of 100 to demonstrate the robust nature of the antiviral action of the combined treatment: two H1N1 [A/PR/8/1934 and A/California/04/2009] and four H3N2 strains [A/Aichi/02/1968, A/Wisconsin/67/2005, A/Texas/50/2012, and A/Hong Kong/8/1968]. AMT treatment resulted in decreases in viral titers (**Figure 1A**) in the case of all strains and decreases in viral gene expression, which were significant in the case of only two out of six IAV strains (**Figure 1B**). OSL treatment had robust and significant reductions in IAV proliferation as measured by PFU assay (**Figure 1A**) and viral RNA synthesis (**Figure 1B**). OSCN^−^ treatment alone led to diminished viral replication and viral gene expression that reached the levels of significance in case of some strains but remained non-significant in case of others (**Figures 1A, B**). When OSCN^−^ and AMT were combined, viral titers were significantly reduced compared to untreated, AMT-treated, or OSCN^−^-treated samples (**Figure 1A**), and viral gene expression levels also showed similar significant differences (**Figure 1B**). No substantial differences in viral replication were found between the combined treatment (OSCN^−^+AMT) and OSL (**Figure 1A**). The applied treatment conditions alone were not cytotoxic to uninfected MDCK cells as was shown by using the cell cytotoxicity MTT assay (**Figure 1C**). Overall, these results suggest that the concerted action of OSCN^−^ and AMT leads to significant improvement in inhibiting viral RNA synthesis and proliferation in MDCK cells as compared to single treatments or no treatment.

### OSCN^−^ and AMT Combination Treatment Inhibits IAV Uncoating and Nuclear Import in Host Cells

**Figure 2:**
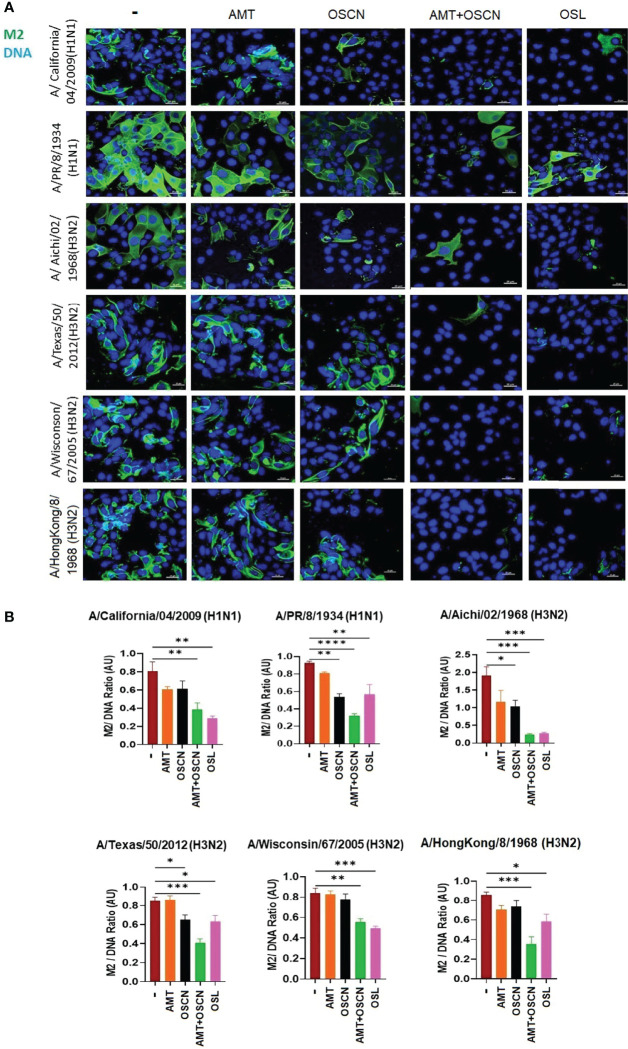
The AMT+OSCN^−^ combination treatment reduces influenza virus uptake into host cells. **(A)** MDCK cells were infected with 100 MOI of the indicated influenza A virus strains and incubated at 4°C for 1 h. Unbound virions were washed out, and MDCK cells were exposed to different treatment conditions for 1 h at 37°C as indicated. Cells were fixed, and the cellular localization of the viral M2 ion channel was detected by immunofluorescence at 3 hpi (green). Cellular DNA was labeled by DAPI (blue). OSL was used as a positive control. Merged images of the viral protein and DAPI-stained nuclei are shown. Images are representative of three independent experiments performed in duplicates (scale bars, 25 μm). The images were acquired at ×60 magnification using confocal microscope. **(B)** Ratios of mean fluorescent intensities (MFI) of the M2 signal divided by the DAPI signal are shown (mean ± SEM). Ratios were measured for each strain using images from three independent experiments n = 3. One-way ANOVA and Tukey’s multiple comparisons tests. AMT, amantadine; AU, arbitrary unit; MOI, multiplicity of infection; M2, M2 ion channel; OSCN^−^, hypothiocyanite; OSL, oseltamivir; MDCK, Madin–Darby canine kidney. *, p < 0.05; **, p < 0.01; ***, p < 0.001; ****, p < 0.0001.

**Figure 3:**
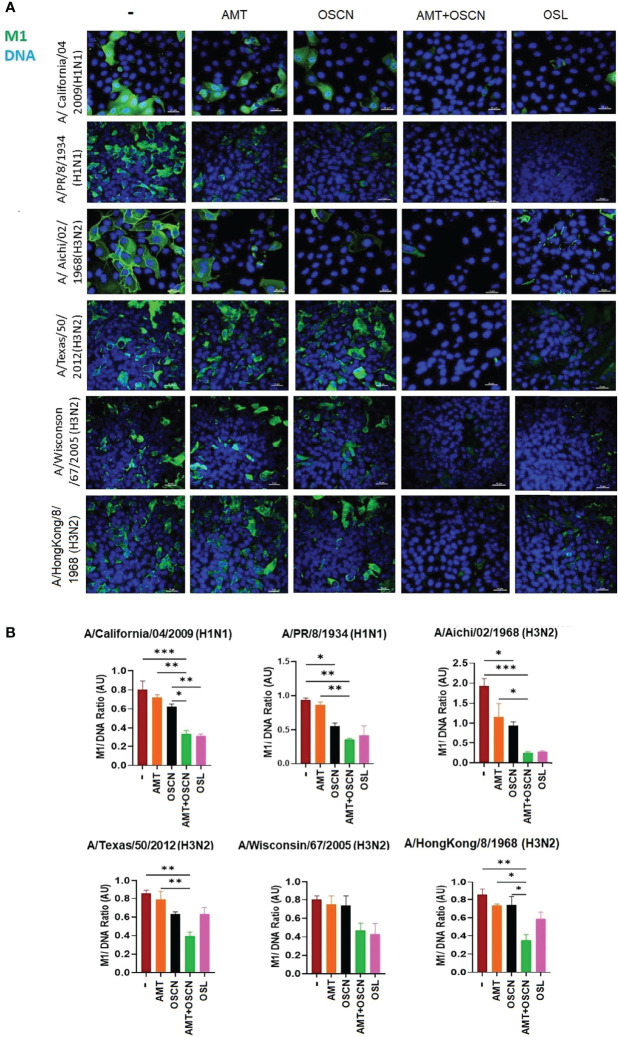
The AMT+OSCN^−^ combination treatment reduces influenza virus uncoating in host cells. **(A)** MDCK cells were infected with 100 MOI of the indicated influenza A virus strains and incubated at 4°C for 1 h. Unbound virions were washed out, and MDCK cells were exposed to different treatment conditions for 1 h at 37°C, as indicated. Cells were fixed, and the cellular location of the viral M1 matrix protein was detected by immunofluorescence at 3 hpi (green). Cellular DNA was labeled by DAPI (blue). OSL was used as a positive control. Merged images of the M1 protein and DAPI-stained nuclei are shown. Images are representative of three independent experiments performed in duplicates (scale bars, 25 μm). The images were acquired at ×60 magnification using confocal microscope. **(B)** Ratios of mean fluorescent intensities (MFI) of the M1 signal divided by the DAPI signal are shown (mean ± SEM). Ratios were measured for each strain using images from three independent experiments n = 3. One-way ANOVA and Tukey’s multiple comparisons tests. AMT, amantadine; AU, arbitrary unit; M1, M1 matrix protein; MOI, multiplicity of infection; OSCN^−^, hypothiocyanite; OSL, oseltamivir; MDCK, Madin–Darby canine kidney. *, p < 0.05; **, p < 0.01; ***, p < 0.001.

**Figure 4:**
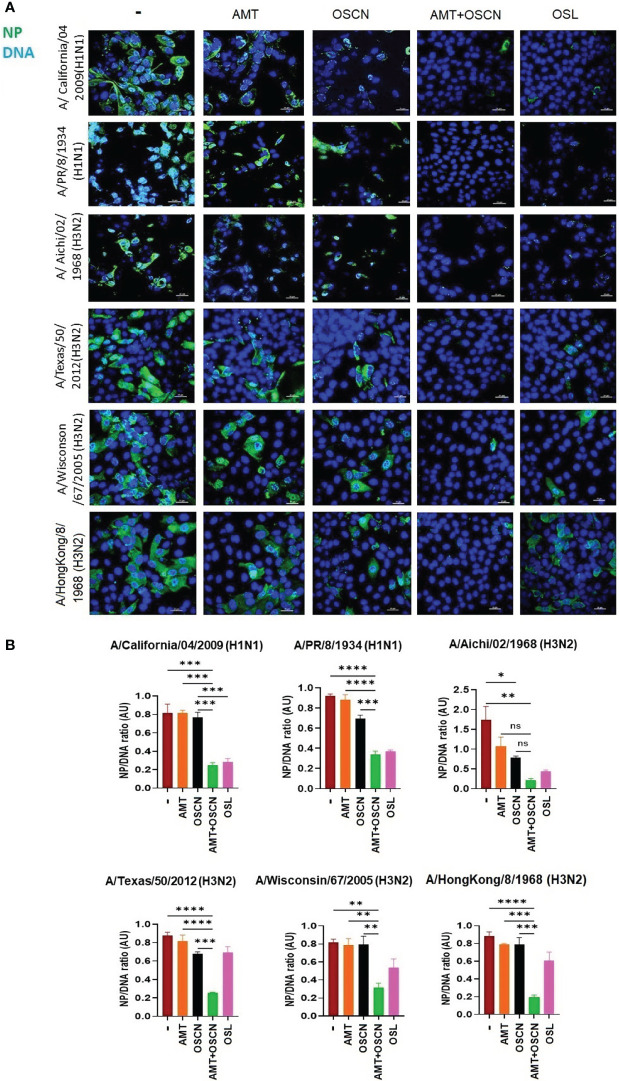
The AMT+OSCN^−^ combination treatment reduces nucleoprotein expression of influenza virus in host cells. **(A)** MDCK cells were infected with 100 MOI of the indicated influenza viral strains and incubated at 4°C for 1 h. Unbound virions were washed out, and MDCK cells were exposed to different treatment conditions for 1 h at 37°C as indicated. Cells were fixed, and the cellular location of the viral nucleoprotein (NP) was detected by immunofluorescence at 6 hpi (green). Cellular DNA was labeled by DAPI (blue). OSL was used as a positive control. Merged images of the viral protein and DAPI-stained nuclei are shown. Images are representative of three independent experiments performed in duplicates (scale bars, 25 μm). The images were acquired at ×60 magnification using confocal microscope. **(B)** Ratios of mean fluorescent intensities (MFI) of the NP signal divided by the DAPI signal are shown (mean ± SEM). Ratios were measured for each strain using images from three independent experiments n = 3. One-way ANOVA and Tukey’s multiple comparisons tests. AMT, amantadine; AU, arbitrary unit; MOI, multiplicity of infection; NP, nucleoprotein; OSCN^−^, hypothiocyanite; OSL, oseltamivir; MDCK, Madin–Darby canine kidney. *, p < 0.05; **, p < 0.01; ***, p < 0.001; ****, p < 0.0001, ns, non-significant.

To further explore whether the combination of AMT and OSCN^−^ affects viral entry, the same six IAV strains were allowed to adhere to MDCK cells for 1 h at 4°C. Unbound virions were washed away, and cells with virions bound to their surface were exposed to the indicated treatment conditions post-binding as described previously (12) under the conditions of **Figure 1**. Cells were fixed 3 h later and stained for the viral M2 ion channel (**Figure 2**) or M1 matrix protein (**Figure 3**), indicative of viral uncoating as before (12). The combined treatment of AMT+OSCN^−^ or OSL alone significantly reduced the M2 immunofluorescent signal (M2/DAPI ratio) as compared to the infected but untreated controls in MDCK cells infected by each of the six IAV strains (**Figures 2A, B**). Similar trends could be observed when the localization of M1 matrix protein as a sign of viral uncoating inside host cells was investigated by immunofluorescence (**Figure 3**). The combined treatment significantly diminished IAV uncoating (M1/DAPI signal ratio) in the case of all IAV strains except A/Wisconsin/67/2005, which had a non-significant reduction (**Figures 3A, B**). Uncoating of the virion content is followed by the translocation of the viral RNA to the host cell’s nucleus and by the synthesis of new viral proteins (21). NP is released upon entry and uncoating into the cytosol and then subsequently translocates to the nucleus (nuclear import) where it participates in the formation of viral ribonucleoproteins that will be transported from the nucleus back to the cytosol (22, 23). Therefore, NP immunofluorescence can be detected in both the cytosol and the nucleus and indicates later stages of the viral life cycle (22). The NP immunofluorescent signal (NP/DAPI ratio) was also significantly reduced by the combined treatment in comparison to untreated cells in the case of all six IAV strains tested (**Figures 4A, B**). The combined treatment showed also a significant reduction of the NP signal compared to the single treatments (AMT and OSCN^−^) in the case of five strains (**Figure 4**). Thus, the combined AMT+OSCN^−^ treatment targets the early steps of influenza replication *in vitro*.

### OSCN^−^ and AMT Combination Treatment Inhibits IAV Replication in Primary Human Bronchial Epithelial Cells

**Figure 5:**
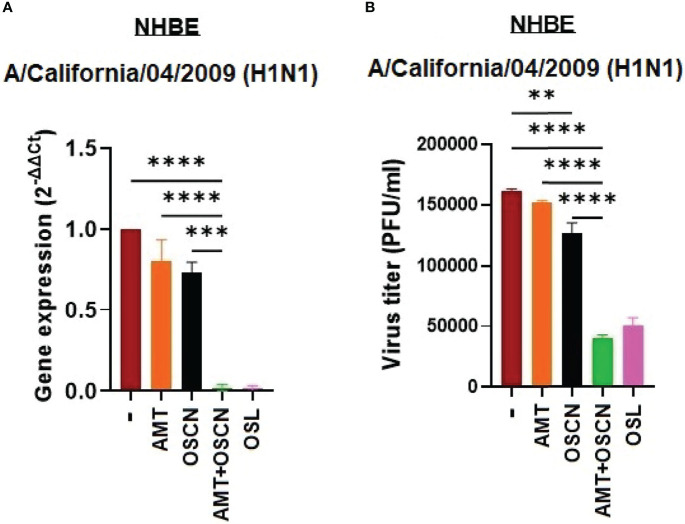
The AMT+OSCN^−^ combination treatment restricts influenza virus replication in primary human bronchial epithelial cells. Primary normal human bronchial epithelial (NHBE) cells were infected with 100 MOI of the A/California/04/09 (H1N1) influenza A virus strain under the indicated conditions for 1 h. **(A)** mRNA expression was measured using real-time qPCR. β-Actin was used as reference. OSL was used as a positive control. **(B)** Viral proliferation was assessed using PFU assay. Data are expressed as mean ± SEM of n = 3 independent experiments performed in triplicates. One-way ANOVA and Tukey’s multiple comparisons tests. AMT, amantadine; MOI, multiplicity of infection; NHBE, normal human tracheobronchial epithelial cells; OSCN^−^, hypothiocyanite; OSL, oseltamivir; PFU, plaque-forming unit. **, p < 0.01; ***, p < 0.001; ****, p < 0.0001.

To confirm the inhibitory action of the combination therapy on IAV replication in physiologically relevant cells, ALI cultures of NHBE cells were infected with only one IAV strain, A/California/04/2009, in the presence of the same treatment conditions as tested before. NHBE cells represent a suitable *in vitro* model of the human respiratory epithelium and, when infected with influenza viruses, express Duox1, produce H_2_O_2_, and generate OSCN^−^ when LPO and SCN^−^ are added exogenously (12, 13, 20, 24, 25). We have previously shown that the A/California/04/2009 virus strain infects NHBE cells and OSCN^−^, reducing its proliferation in an NHBE-generated H_2_O_2_-dependent manner (12). The combination of AMT and OSCN^−^ led to significantly reduced viral RNA synthesis (**Figure 5A**) and IAV replication (**Figure 5B**) in NHBE cells as compared to untreated cells or single treatments. Single treatments did not show significant efficacy (**Figure 5**). These data show that the combination treatment inhibits IAV replication in both MDCK cells and NHBE cells.

### The AMT+OSCN^−^ or OSCN^−^+AMT+OSL Treatments Inhibit the Replication of AMT-Resistant IAV Strains

**Figure 6:**
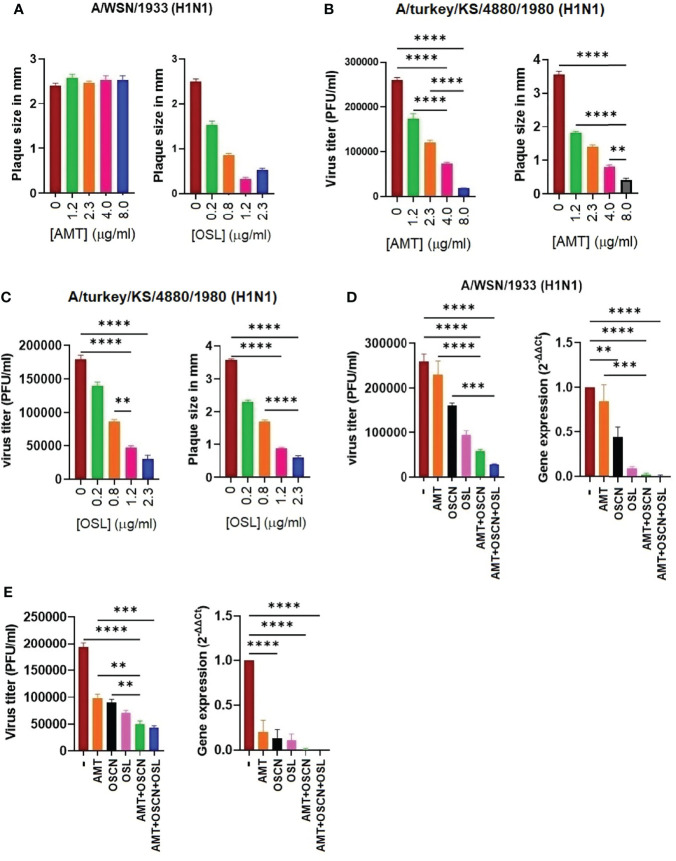
A triple combination therapy including AMT, OSCN^−^, and OSL, is superior to dual and single drug regimens against amantadine-sensitive and amantadine-resistant influenza A strains *in vitro*. Confluent MDCK cell monolayers in 6-well plates were infected with 100 MOI of the **(A)** A/turkey/Kansas/4880/1980 (H1N1) (AMT-susceptible strain) or **(B, C)** the A/WSN/1933 (H1N1) (AMT-resistant) IAV strains. Cells were incubated with overlay media containing the indicated antiviral drugs and their combinations. The plaque numbers or the average plaque sizes measured in diameter were determined at 3 dpi. Mean ± SEM, three independent experiments. The efficacy of the triple combination treatment consisting of OSCN^−^, AMT, and OSL on viral replication (plaque assays) and viral gene expression (qPCR) is shown for **(D)** the A/WSN/1933 (H1N1) and **(E)** A/turkey/Kansas/4880/1980 (H1N1) IAV strains. [AMT] = 15 μg/ml, [OSL] = 5 μg/ml. β-Actin was used as a housekeeping gene. Data are presented from three individual experiments (n = 3). One-way ANOVA and Tukey’s multiple comparisons tests. AMT, amantadine; IAV, influenza A virus; OSCN^−^, hypothiocyanite; MOI, multiplicity of infection; OSL, oseltamivir; PFU, plaque-forming unit; MDCK, Madin–Darby canine kidney. **, p < 0.01; ***, p < 0.001; ****, p < 0.0001.

We next aimed at investigating whether OSCN^−^ alone or in combination with AMT or AMT+OSL is efficient in preventing the replication of an AMT-resistant IAV strain. Since all six IAV strains tested showed various levels of susceptibility to AMT treatment *in vitro* (**Figures 1**–**4**), we evaluated A/WSN/33, which is a strain with well-documented AMT resistance, which has been linked to molecular changes associated with resistance identified as single-nucleotide changes leading to corresponding amino acid substitutions in one of four critical sites, amino acids 26, 27, 30, and 31, in the transmembrane region of the M2 protein (26). We confirmed the AMT resistance of the A/WSN/1933 (H1N1) strain by plaque size measurements (**Figure 6A**), viral RNA synthesis, and proliferation (**Figure 6D**). In contrast, the A/turkey/Kansas/4880/1980 (H1N1) virus strain, which was shown to be susceptible to OSCN^−^ in our prior report (15), is highly susceptible to AMT as also assessed by PFU titers and sizes (**Figure 6B**). Both strains are susceptible to OSL (**Figures 6A, C**). After having characterized the drug resistance of these two strains, next, we evaluated the effect of the AMT+OSCN^−^ double treatment and the triple combination of AMT, OSCN^−^, and OSL on viral gene expression and proliferation using quantitative PCR and plaque formation using MDCK cells. We observed pronounced therapeutic effects of both the double and triple treatment combinations in the case of both strains as compared to the single-agent therapies or the “no treatment” control (**Figures 6D, E**). However, the combined effect of the TCAD regiment was greater than that of any double combination tested. These results demonstrate that combining OSL with the dual combination provides additional synergy.

### OSCN^−^+AMT Combination Treatment Protects Mice Against Lethal Infection by an H1N1 IAV Strain

**Figure 7:**
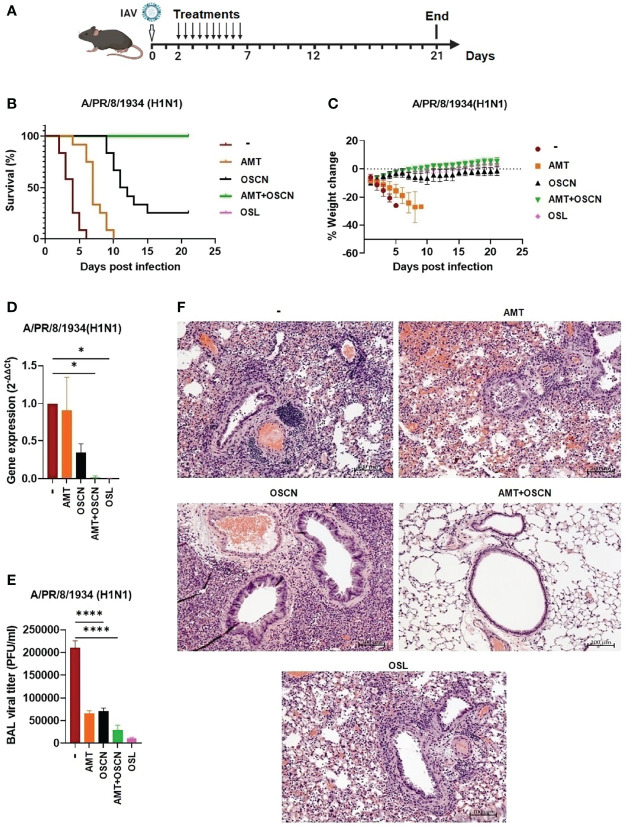
The AMT+OSCN^−^ combination treatment cured mice from lethal H1N1 influenza A virus infection. C57BL/6 mice were infected intranasally with 150 PFU of A/PR/8/34 (H1N1). Mice were treated with the indicated antiviral drugs and their combinations for 5 days (2–7 dpi) by oral gavage (two doses daily). In the untreated “placebo” group, animals received the equivalent volume of distilled water. Animals were observed until 21 dpi. **(A)** Experimental scheme. **(B)** Mortality was measured and presented as Kaplan–Meier survival curves. **(C)** Weight changes are presented as percentages of the initial body weight of the animals (6 mice per group, two independent experiments). At 3 dpi, BAL from infected mice treated as indicated was collected, and BAL cells and cell-free supernatants were separated by centrifugation. **(D)** Viral gene expression in BAL cells was measured by qPCR and normalized on the values of the infected but untreated group. **(E)** Viral titers in cell-free BAL supernatants were determined by PFU assays. Data are expressed as mean ± SEM of n = 3 independent experiments that each used 3 animals per group. **(F)** Histopathological images of H&E-stained, fixed lung tissue sections of infected mice treated as indicated (3 dpi). Magnification, ×10; scale bars, 100 μm. AMT, amantadine; BALF, bronchoalveolar lavage fluid; OSCN^−^, hypothiocyanite; OSL, oseltamivir; PFU, plaque-forming unit. *, p < 0.05; ****, p < 0.0001.

To demonstrate the therapeutic efficacy of the combination of AMT and OSCN^−^ on airway infection elicited by an H1N1 IAV strain, C57BL/6 mice were intranasally infected with a lethal dose (150 PFU) of the A/PR/8/1934 virus strain. All drugs were administered in equally divided doses (twice a day) by oral gavage for 5 days in a row starting 2 days after IAV infection (**Figure 7A**). All drug concentrations investigated were clinically relevant doses and were used for murine efficacy studies (18, 19). Five treatment conditions were used; OSL was administered at a daily dose of 25 mg/kg and AMT at a dose of 46 mg/kg. OSCN^−^ was generated *in vitro* in a cell-free system as described previously (18, 19), the same way as in the *in vitro* assays (**Figures 1**–**6**). Mice infected with IAV but only treated with solvent (placebo group) all succumbed to the infection at 6 dpi (**Figure 7B**). AMT treatment delayed the disease progression, but all AMT-treated mice died at 10 dpi (**Figure 7B**). When OSCN^−^ was used alone, it rescued some animals from death at 21 dpi (**Figure 7B**). Impressively, the combined (AMT+OSCN^−^) and OSL treatments provided 100% protection and resulted in complete survival of all the animals at 21 dpi (**Figure 7B**). Untreated or AMT-treated mice suffered significant weight losses due to disease progression (**Figure 7C**). OSCN^−^-treated mice demonstrated some temporary weight loss, while animals treated with OSL or the AMT+OSCN^−^ combination did not lose weight (**Figure 7C**). Treatment efficacy on viral replication was also tested by using BAL samples from different treatment groups by performing viral plaque assay on the BAL supernatants and viral qPCR in BAL cells. Both the AMT+OSCN^−^ and OSL-treated groups showed significantly lower viral titers and gene expression levels as compared to the placebo (untreated) group at 5 dpi (**Figures 7D, E**). Histopathological evaluation of the lung in the placebo, AMT, OSCN^−^, and OSL monotherapy groups at 5 dpi (3 days posttreatment) showed congestion and destruction of the bronchial/bronchiolar epithelium and alveolar cells and associated mixed inflammatory infiltrate and hemorrhage. The bronchiolar epithelium was often hyperplastic, indicating bronchiolar epithelial regeneration (**Figure 7F**). However, the combined AMT+OSCN^−^ treatment revealed fewer pathological effects resembling a healthy lung (**Figure 7F**). Thus, we conclude that the combined treatment of OSCN^−^ and AMT is effective against the lethal infection by an H1N1 IAV strain (A/PR/8/1934) in this murine model.

### OSCN^−^+AMT Combination Treatment Protects Mice Against a Lethal Infection by an H3N2 IAV Strain

**Figure 8:**
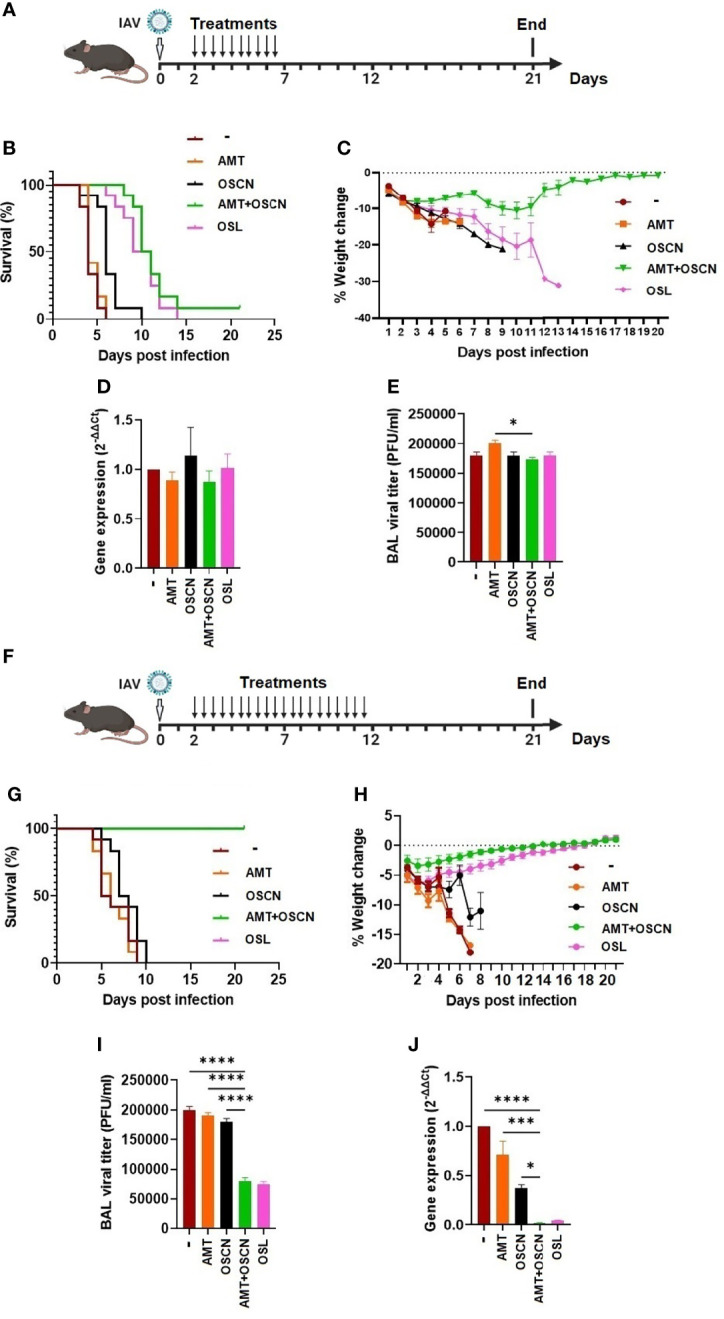
The AMT+OSCN^−^ combination treatment cured mice of lethal H3N2 influenza A virus challenge. **(A–E)** C57BL/6 mice were infected intranasally with 3,000 PFU of the A/Hong Kong/8/1968 (H3N2) IAV strain. Animals were treated with the indicated antiviral drugs and their combinations for 5 days (2–7 dpi) by oral gavage (two doses daily). In the untreated “placebo” group, animals received the equivalent volume of distilled water. Animals were observed until 21 dpi. **(A)** Experimental timeline for panels **(A–E)**. **(B)** Mortality was measured and presented as Kaplan–Meier survival curves, and **(C)** weight changes are presented as percentages of the initial body weight of the animals (6 mice per group, two independent experiments). At 3 dpi, BAL from infected mice treated as indicated was collected, and BAL cells and cell-free supernatants were separated by centrifugation. **(D)** Viral gene expression in BAL cells was measured by qPCR and normalized on the values of the infected but untreated group. **(E)** Viral titers in cell-free BAL supernatants were determined by PFU assays. Data are expressed as mean ± SEM of n = 3 independent experiments that each used 3 animals per group. **(F–J)** Similarly to panels A–E, C57BL/6 mice were infected intranasally with 3,000 PFU of A/HK/1968 (H3N2) IAV. Animals were, however, treated with the indicated antiviral drugs and their combinations for a longer period of time, 10 days (2–12 dpi), by oral gavage (two doses daily). In the untreated “placebo” group, animals received the equivalent volume of distilled water. Animals were observed until 21 dpi. **(F)** Experimental timeline for panels **(F–J)**. **(G)** Mortality, **(H)** weight changes, **(I)** BAL cell viral gene expression, and **(J)** BAL supernatant viral titers were determined as indicated previously. AMT, amantadine; BALF, bronchoalveolar lavage fluid; OSCN, hypothiocyanite; OSL, oseltamivir; PFU, plaque-forming unit. *, p < 0.05; ***, p < 0.001; ****, p < 0.0001.

As the AMT+OSCN^−^ combination treatment was successful at rescuing mice from lethal infection (**Figure 7**), we explored whether this effect would also occur in mice lethally infected with an H3N2 IAV strain that also causes seasonal epidemics and infects immunocompromised patients in hospitals (27–30). Mice were intranasally infected with a lethal dose of the A/Hong Kong/8/1968 IAV strain that is also susceptible to OSCN^−^ (15), AMT, and OSL (**Figures 1**–**4**). Mice were infected on day 0 and subjected to the same five treatment conditions 2–7 dpi (**Figure 8A**) as PR8 was before (**Figure 7**). As expected, all mice succumbed to the infection at 6 dpi but were unaffected by AMT treatment (**Figure 8B**). While OSCN^−^ treatment slightly delayed this response, all animals expired at 10 dpi (**Figure 8B**), similar to mice infected with the H1N1 IAV strain (**Figure 7B**). Interestingly, the AMT+OSCN^−^ combination treatment or the OSL monotherapy did not prevent the occurrence of mortality but only delayed its onset (**Figure 8B**). All the animals succumbed to the infection at 14 dpi despite the 5-day-long OSL treatment, and only one animal remained alive at 21 dpi in the combination treatment group (**Figure 8B**). This is in contrast to the results obtained using the H1N1 strain (**Figure 7B**). All animals experienced significant weight loss before succumbing to the infection (**Figure 8C**). Interestingly, the differences seen in mortality and weight loss between the treatment groups were not clearly reflected in viral titers (**Figure 8D**) and viral RNA levels (**Figure 8E**) in BAL samples collected at 3 dpi.

In an effort to improve the efficacy of the combination treatment against the H3N2 IAV strain used, we decided to prolong the treatment period from 5 to 10 days. Thus, mice were intranasally infected with the same lethal dose of the A/Hong Kong/8/1968 (H3N2) IAV strain and treated in the same ways as before between 2 and 12 dpi (10-day duration) (**Figure 8F**). Impressively, mice in both the AMT+OSCN^−^ combination therapy and OSL treatment groups survived and regained their initial weight after completing the whole 21-day-long course of the experiment (**Figures 8G, H**). The monotherapies remained ineffective (**Figures 8G, H**).

BAL samples taken from surviving mice at 10 dpi (8 days posttreatment) revealed significantly lower viral titers (**Figure 8I**) and RNA levels (**Figure 8J**) in the combined therapy and OSL-treated groups compared to the placebo or the AMT or OSCN^−^ monotherapy groups, further confirming the beneficial effects of the combined and OSL treatments.

Overall, AMT+OSCN^−^ combination therapy and OSL monotherapy protected mice from lethal infections by both H1N1 and H3N2 IAV strains. Prolonging the treatment period proved to be successful in improving poor therapeutic effects.

## Discussion

Several studies have reported that combining anti-influenza drugs can enhance the synergy in inhibiting viral replication *in vitro* or *in vivo* (31–33). The rationale behind this study was to test whether OSCN^−^ alone has antiviral action against influenza *in vivo* or can increase the efficacy of other antiviral drugs. Furthermore, knowing that the majority of influenza A strains have lower susceptibility to the current, Food and Drug Administration (FDA)-approved anti-influenza drugs, their possible combination with new antivirals such as OSCN^−^ could synergistically improve their therapeutic action.

Combination treatment of AMT and OSCN^−^ was highly efficacious in reducing weight loss and death in mice infected with A/PR/8/1934 and A/Hong Kong/8/1968 influenza viruses in our study. The therapeutic efficacy of the combined regimen was superior to that of treatment with a single drug, and the enhanced potency of AMT combined with OSCN^−^ *in vivo* confirmed the synergy shown *in vitro*. The contribution of AMT to the therapeutic efficacy of a combined regimen in mice and *in vitro* indicates the synergistic antiviral effects of two independent therapeutic approaches. Furthermore, the therapeutic effect of TCAD treatment against A/WSN/1933 (AMT-resistant strain) and A/turkey/Kansas/4880/1980 (H1N1) (AMT-susceptible strain) was observed when OSL was added to the AMT+OSCN^−^ combination. Generally, the dosing regimens used in our *in vitro* cell cultures and murine model were depicted to provide similar drug exposure to those carried out in humans and were all according to pharmacokinetic parameters achieved for used drugs in mice and humans.

Although the drug toxicity was not directly measured in this study, we have assessed potential drug toxicity by single agents and found that the anti-influenza drugs do not cause detectable toxicity *in vitro* (**Figure 1C**). Toxicity is unlikely to become a clinical issue when considering potential, future, human clinical tests, as AMT and OSL have been used in humans for years, and OSCN^−^ is present in the human body at the concentrations used in this study. In addition, the drug concentrations of AMT and OSL used in this study were 5- to >300-fold below the 50% lethal concentrations of each single treatment (34–36). The fact that the single treatment of AMT at its highest dose (46 mg/kg) cannot improve the survival rate in mice, while all mice survived in the combined group, and the fact that all mice in the combined group gained weight during the course of treatment demonstrates that any toxic side effects did not affect our parameters of therapeutic efficacy (mortality and weight loss).

In our *in vitro* model, the triple combination treatment was effective against the AMT-susceptible [A/turkey/Kansas/4880/1980 (H1N1)] and AMT-resistant strain (A/WSN/1933) IAV strains as well. AMT, OSL, and OSCN^−^ target different points of the virus replication cycle: M2 ion channel, neuraminidase, and hemagglutinin [potentially (12)], respectively. The advantage of any combination treatment is that there is not only one but also two or more targets increasing the chances to be clinically effective and decreasing the chances of emerging drug resistance. Furthermore, OSCN^−^ (or SCN^−^ also added as part of the OSCN^−^-generating solution) likely has an additional, beneficial anti-inflammatory effect too since it could diminish neutrophil-mediated, oxidative tissue damage that has been associated with IAV-induced lung injury (37, 38). Interactions among NA, M2, and hemagglutinin have been reported to affect susceptibilities to AMT and OSL, respectively (39, 40), and could represent one possible explanation for the synergistic effects of the tested antiviral drugs. Previous studies have reported that HA–M2 and HA–NA interactions can affect the susceptibility to AMT and OSL, respectively (41, 42). The presence of the third compound is important for the synergistic antiviral efficacy of the combined treatment. In our previous study, OSCN^−^ demonstrated an inhibitory effect on influenza viral attachment and entry with several proposed mechanisms of action, which remain to be explored (12). These data indicate that the combined treatment may provide broad-spectrum antiviral activity against circulating IAVs, including strains that are resistant to either class of antivirals. Currently, most influenza A strains (99%) are resistant to either the adamantanes or OSL, but not to both (9), and therefore are expected to be susceptible to the combined treatment. At the present time, fast diagnostic tests are not available to evaluate the susceptibility profile of influenza viruses in patients in real time, and hence clinicians do not often have the needed information based on which to decide the use of the appropriate antiviral treatment. The accessibility of a broad-spectrum anti-influenza medication effective against most of the currently circulating strains would be of maximum clinical benefit. Since OSCN^−^ has antimicrobial activities against a wide range of pathogens [reviewed by us here (43)] including respiratory viral and bacterial pathogens, results presented in this work also indicate that combining OSCN^−^ with other drugs represents a potential therapeutic option for several additional infectious agents beyond IAVs.

This study focused on the combination of OSCN^−^ with other antiviral drugs, although data in this manuscript also demonstrate that OSCN^−^ alone has beneficial effects against PR8 infection in mice. While mortality was not prevented by OSCN^−^ alone, we also selected a relatively low dose of OSCN^−^ (100 μl volume per oral gavage) for our experiments so that a potential synergistic effect by combining it with AMT could be observed. It remains to be determined whether OSCN^−^ alone, at doses higher than used here, can further improve the outcomes of lethal influenza A infection in mice.

While we have not performed an *in vivo* infection in mice with the AMT-resistant IAV strain A/WSN/1933 (H1N1), we expect that the OSCN^−^+AMT combination treatment would turn out to be less efficient than against the two viruses tested. In our view, this would be mainly due to the presence of only one, potentially efficient, antiviral drug, OSCN^−^. Since the TCAD treatment further inhibited *in vitro* proliferation of the A/WSN/1933 (H1N1) virus strain, we believe the TCAD treatment would further improve the outcomes of an *in vivo* infection beyond the double treatment.

Our results indicate that a combined anti-influenza regimen including AMT, OSCN^−^, and OSL could represent an innovative therapeutic option for the treatment of severe outcomes of seasonal and pandemic influenza infections. We intentionally chose lethal doses of the influenza viruses tested *in vivo* because they represent an appropriate animal model for those, mainly immunocompromised, human patients who are hospitalized or even die following influenza infection worldwide. While influenza infections leading to death or admittance to the hospital make up only a minor portion of the total influenza cases, they represent the clinically most severe symptoms and need effective treatment options the most (44–47). Most of our outcomes in this study support the combined hypothesis, which declares that for any type of susceptible or resistant circulating influenza virus, at least two, and in some combinations all three, compounds in combined treatment will be effective. Moreover, the combined treatment seems to diminish drug resistance and hence could be a highly active antiviral therapy for seasonal and pandemic influenza. The pharmacokinetics, safety, hepatic metabolism, and plasma distribution of AMT and OSL as a monotherapy are well-acknowledged, and it is not predicted that co-administration of these compounds will cause harmful side effects to patients as compared to the administration of the single agent. Additionally, the AMT and OSL combination has been tested in humans without treatment complications (17).

In summary, we provide data for the successful use of the OSCN^−^+AMT combination treatment to prevent lethal influenza A virus infection in mice. Future clinical trials would be needed to evaluate the efficacy and safety of the AMT and OSCN^−^ combination against seasonal and pandemic influenza A virus strains.

## Data Availability Statement

The raw data supporting the conclusions of this article will be made available by the authors, without undue reservation.

## Ethics Statement

The animal study was reviewed and approved by the Animal Care and Use Committee at the University of Georgia

## Author Contributions

NA and BR designed the experiments. NA conducted most of the experiments and performed data analysis. DS helped with performing mouse infection studies. TN performed histological analysis. NA wrote the original draft of the manuscript. NA, DS, TN, ZR, RAT, and BR revised and edited the manuscript. BR also performed data analysis. BR and RAT acquired funding and oversaw the project. All the authors read and approved the final version of the manuscript.

## Funding

This work was supported by the National Institutes of Health (to BR and RAT, R01AI146857).

## Author Disclaimer

The content is solely the responsibility of the authors and does not necessarily represent the official views of the National Institutes of Health.

## Conflict of Interest

The authors declare that the research was conducted in the absence of any commercial or financial relationships that could be construed as a potential conflict of interest.

## Publisher’s Note

All claims expressed in this article are solely those of the authors and do not necessarily represent those of their affiliated organizations, or those of the publisher, the editors and the reviewers. Any product that may be evaluated in this article, or claim that may be made by its manufacturer, is not guaranteed or endorsed by the publisher.

## Abbreviations

ALI, air–liquid interface; AMT, amantadine; BALF, bronchoalveolar lavage fluid; dpi, days post-infection; Duox, dual oxidase; HA, hemagglutinin; hpi, hours post-infection; IAV, influenza A virus; i.n., intranasally; LPO, lactoperoxidase; MDCK, Madin–Darby canine kidney; MOI, multiplicity of infection; NA, neuraminidase; NAI, neuraminidase inhibitors; NHBE, normal human bronchial epithelium; OSCN^−^, hypothiocyanite; OSL, oseltamivir; PFU, plaque-forming unit; PR8, A/Puerto Rico/8/1934; RT-PCR, reverse transcription–PCR; SCN^−^, thiocyanate; TCAD, triple combination of anti-influenza drugs; WT, wild-type.
